# Ketamine in psychiatry: a systematic review of clinical applications, safety considerations, and emerging challenges

**DOI:** 10.3389/fpsyt.2026.1949261

**Published:** 2026-10-01

**Authors:** Matthew M. Kinney, Zackary L. Bowers

**Affiliations:** 1 College of Human Medicine, Michigan State University, East Lansing, MI, United States; 2 Department of Psychology, Alma College, Alma, MI, United States

**Keywords:** esketamine, ketamine, major depressive disorder, NMDA receptor antagonists, off-label use, recreational use, telehealth, treatment-resistant depression

## Abstract

Ketamine has progressed from solely being an anesthetic agent to a drug of rising clinical and research interest due to its distinctive pharmacological properties. Its dissociative properties and rapid-acting antidepressant effects have made it a prominent area of investigation across many medical and psychiatric applications. This systematic review examines ketamine’s history, mechanisms of action, clinical uses, and significant challenges. A systematic search of PubMed, SCOPUS, PsycINFO, and other sources, generated peer-reviewed publications that addressed the history of ketamine, its pharmacology, clinical applications, recreational use, safety considerations, and societal consequences. Overall, 106 articles were identified for inclusion in this study. At the receptor level, ketamine is an antagonist of NMDA receptors that triggers a number of downstream effects ranging from heightened glutamate transmission, AMPA receptor activation, increased BDNF expression, synaptogenesis, and mTOR pathway signaling. At the network level, it alters activity within large-scale brain circuits including the default mode network, affecting cognition, mood, and perception. Clinically, ketamine is used for anesthesia, treatment-resistant depression, and pain, with off-label uses for PTSD, anxiety disorders, and bipolar disorder. The review also examines current challenges related to ketamine’s recreational use, associated risks, sociocultural factors, and key clinical and ethical considerations, including safety, monitoring, and telehealth prescribing of oral and sublingual formulations. It integrates previously fragmented research from anesthesiology, neuroscience, pharmacology, psychiatry, and public health, consolidates clinical and ethical guidance, identifies critical gaps in current knowledge, and proposes directions for future research.

## 1 Introduction

Ketamine has played a multifaceted and changing role across both medical and recreational contexts. Initially developed as an anesthetic, ketamine’s unique pharmacological properties—most notably its dissociative effects and rapid-acting antidepressant potential have made it a subject of growing scientific and clinical interest. Over the decades, its use has expanded from surgical anesthesia, use on battlefields, to the treatment of depression, chronic pain, and other psychiatric disorders. Simultaneously, ketamine has also become a popular substance of abuse, especially in nightclubs and party settings, where it is valued for its hallucinogenic and euphoric effects.

This systematic literature review examines ketamine across its historical development, pharmacological mechanisms of action, and clinical applications, including both approved and off-label uses in psychiatric conditions. It also addresses ketamine’s recreational use and abuse, associated risks, sociocultural factors, and key clinical and ethical considerations such as safety, monitoring, and telehealth prescribing. In addition, the review highlights major challenges in clinical practice, including translating ketamine’s rapid antidepressant effects into sustained long-term benefits, balancing efficacy with safety and abuse liability, and navigating regulatory and access-related barriers. Given ketamine’s position at the intersection of anesthesiology, psychiatry, neuroscience, pharmacology, public health, ethics, and policy, and its rapidly evolving evidence base, this review provides a valuable synthesis of previously fragmented multidisciplinary research. It evaluates ketamine’s efficacy and safety across a range of indications, informs evidence-based clinical decision-making, and weighs therapeutic benefits against social, ethical, and public health risks. By articulating professional standards and ethical considerations for clinicians and offering insights relevant to policymakers, this review serves as an authoritative, evidence-based resource for clinicians, researchers, patients, and society; it also identifies significant gaps in current knowledge and suggests future directions for research.

## 2 Methods

In order to capture ketamine’s wide-ranging clinical, mechanistic, and sociocultural roles, we conducted a structured literature search, following the Preferred Reporting Items for Systematic reviews and Meta-Analyses (PRISMA) guidelines, using a transparent and reproducible review framework (1). Our aim in designing this article was to integrate both historical and contemporary evidence regarding ketamine’s pharmacology, clinical applications, recreational use, and policy implications. Specifically, we searched the following databases: PubMed, SCOPUS, Embase, PsycINFO, ProQuest, and Google Scholar for ketamine research that was published between January 2000 and June 2026. Foundational studies published before 2000, including Collingridge (1987) (2), Domino (1980) (3), Anis (1983) (4), among others, were also included to contextualize the historical development of ketamine. The search involved keywords related to ketamine’s pharmacology, clinical indications, and sociocultural aspects, including “ketamine,” “esketamine,” “arketamine,” treatment-resistant depression, “NMDA antagonist,” “anesthesia,” “depression,” off-label uses of ketamine, “post-traumatic stress disorder,” “anxiety,” “bipolar disorder,” “pain,” “epilepsy,” “recreational use,” “club drug,” “psychedelic therapy,” “telehealth,” “policy,” and “regulation.” Search queries utilized truncation symbols and Boolean operators in an effort to ensure comprehensive coverage and retrieval of the relevant literature.

While a structured search strategy was employed following the PRISMA guidelines, this systematic review may not have capture every relevant study. Included studies vary in design, population, and context, which may have limited the ability to draw uniform conclusions across all domains.

### 2.1 Publication eligibility criteria

Publications were eligible if they were peer-reviewed studies involving human subjects, including clinical trials, observational studies, reviews, systematic reviews, or meta-analyses. Preclinical studies were also considered for inclusion if they provided insights into biological or translational psychiatry mechanisms relevant to ketamine. Only English-language publications were considered. Moreover, case reports were excluded unless clinically or historically significant. Further, conference abstracts and non-English articles were also excluded.

### 2.2 Screening and extraction of articles

Titles and abstracts of the retrieved literature were screened for relevance by two reviewers. Articles that were found not to be relevant were excluded. This was followed by a full-text reading of the articles of the remaining articles. Any discrepancies were resolved through discussion. Articles were included if they were relevant to at least one of the predefined domains: historical context, mechanism of action, on-label and off-label clinical applications, recreational use, ethical considerations, or policy/regulatory perspectives.

### 2.3 Assessment of methodological quality and risk of bias

Risk of bias and methodological quality were assessed using the study-specific Joanna Briggs Institute (JBI) Critical Appraisal Tools. Given the diverse range of designs included—spanning clinical trials, observational studies, case reports, mechanistic studies, and secondary reviews—we used these checklists to pinpoint specific methodological limitations rather than calculate a single pooled score. Key appraisal domains included sampling, randomization and blinding, outcome measurement reliability, follow-up retention, and potential bias. Rather than excluding studies based on quality scores, these assessments guided how heavily we weighted individual findings in our overall analysis.

## 3 Results

A total of 11,745 records were identified from the database searches, from January 2000 to June 2026. Before screening, 3,722 duplicate records were identified and removed. Subsequently, 8,023 records were screened, with 7,513 non-relevant records being excluded. The remaining 510 records were retrieved and underwent full-text review. Overall, 106 records were identified for inclusion in this study, with 404 records being excluded as they did not fit the predefined publication eligibility criteria. The study selection process is summarized in the PRISMA flow diagram (**Figure 1**).

**Figure 1:**
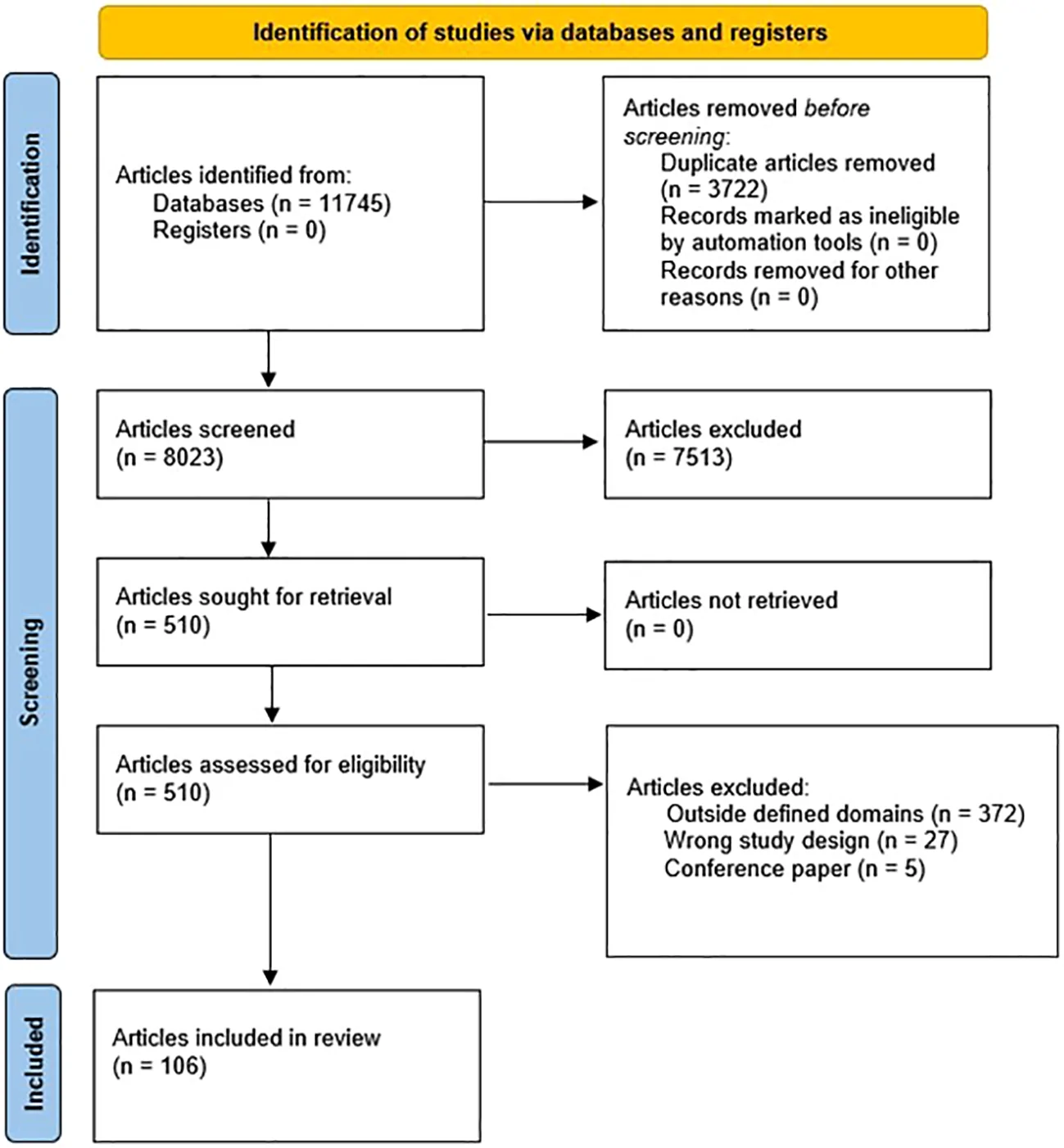
PRISMA 2020 flow diagram of the study selection process.

### 3.1 Characteristics of included studies

The systematic search yielded 106 studies meeting full eligibility criteria for synthesis. This final sample spans psychiatric, neurological, mechanistic, and public health investigations, incorporating randomized controlled trials, observational studies, case reports, preclinical work, and secondary literature (systematic reviews and meta-analyses).

Among the clinical studies, primary evaluations focused on ketamine and esketamine for treatment-resistant depression, major depressive disorder with acute suicidal ideation or behavior, post-traumatic stress disorder, bipolar depression, and anxiety disorders. While randomized trials focused heavily on efficacy, safety, and response durability, observational data provided insight into real-world effectiveness, long-term maintenance, and implementation.

Beyond clinical trials, the included literature covered ketamine’s core neurobiological mechanisms—specifically NMDA receptor modulation, glutamatergic signaling, synaptic plasticity, neurotrophic pathways, and functional network alterations—alongside studies on recreational use patterns, adverse events, regulatory policy, and risk mitigation. Given the high degree of clinical and methodological heterogeneity across study designs, populations, and outcomes, we synthesized the findings narratively.

### 3.2 Methodological quality and risk of bias

Methodological quality varied across the included studies. Randomized controlled trials generally carried the lowest risk of bias, benefiting from structured control designs, clear randomization, and standardized outcome measures. However, common limitations included small sample sizes, brief follow-up windows, and functional unblinding caused by ketamine’s overt psychoactive effects. Observational studies were more vulnerable to selection bias, unmeasured confounding, and inconsistent dosing or treatment protocols across different clinical settings. While case reports and small uncontrolled studies offered early insights into emerging ketamine applications, their lack of control groups inherently limits their generalizability. Secondary reviews generally demonstrated a low risk of bias, whereas mechanistic studies carried a higher or more variable risk of bias driven by small sample sizes, exploratory designs, and varied measurement tools.

## 4 History of ketamine

Ketamine, a Schedule III controlled substance, has a history spanning more than five decades. In the 1950s, Parke-Davis researchers developed phencyclidine (PCP) as an anesthetic; however, its prolonged psychotomimetic effects (prolonged delirium, heightened excitement) limited clinical utility. In 1962, Calvin Stevens synthesized ketamine, a PCP derivative with fewer psychotomimetic effects. Early human studies demonstrated potent anesthetic and analgesic properties with preservation of respiratory function and rapid recovery, leading to its classification as a “dissociative anesthetic” (3, 5–8). First used as a veterinary anesthetic in Belgium in 1963, it was subsequently introduced for human use as ketamine hydrochloride (Ketalar) in 1969. In 1970, racemic ketamine (Ketalar) was approved by the FDA for anesthesia (7). Its ability to preserve cardiovascular and respiratory function and its short half-life made ketamine particularly valuable for field anesthesia, including in wounded soldiers during the Vietnam War (5). Concurrently, its psychoactive effects contributed to recreational misuse in Vietnam and the United States (9, 10), ultimately leading to its classification as a Schedule III controlled substance in 1999 (7).

From the 1960s through the 1980s, ketamine use expanded beyond the operating room to emergency, trauma, and procedural settings. Concurrent advances in neuroscience established the role of glutamatergic N-methyl-D-aspartate (NMDA) receptors in synaptic plasticity, memory, and cognition (2, 11, 12). The subsequent recognition of ketamine as an uncompetitive NMDA receptor antagonist provided important insights into the mechanisms underlying hyperalgesia, schizophrenia, and cognitive function (4, 7, 12). Since the 1990s, evidence of rapid stress disorder effects at subanesthetic doses has prompted extensive investigation of ketamine for major depressive disorder, treatment-resistant depression (13–19), anxiety disorders (20–22), post-traumatic stress disorder (13, 23, 24), and bipolar disorder (25–28). Although ketamine has also been studied for numerous nonpsychiatric conditions, these applications are beyond the scope of the present review.

## 5 Mechanisms of action (depression)

### 5.1 Receptor level

Ketamine is a chiral molecule; it has two enantiomers, (R)-ketamine and (S)-ketamine. (S)-ketamine exhibits around four times greater affinity for the NMDAR compared to (R)-ketamine. Studies have demonstrated that (R)-ketamine has a longer half-life compared to (S)-ketamine, which is likely why it produces superior and longer-lasting antidepressant effects. (S)-ketamine is used more frequently in clinical settings than (R)-ketamine primarily due to the higher potency at NMDAR and more predictable pharmacokinetics with a shorter half-life (29–31). It is currently accepted that ketamine has multiple mechanisms of action that likely act in a complementary fashion.

#### 5.1.1 Glutamatergic NMDA receptors

The most studied mechanism of action for ketamine is inhibition of NMDA receptors (NMDARs) in the brain. NMDARs are glutamatergic ion channel receptors that form a heterotetrameric protein complex (32). NMDA receptor activation requires L-glutamate binding, co-agonist binding of glycine or D-serine, and membrane depolarization to relieve the magnesium block in the channel pore. When the NMDA receptor channel opens, it allows calcium and sodium ions to enter the neuron and potassium ions to exit, thereby contributing to a depolarizing excitatory signal. The generated depolarizing excitatory signal is responsible for the signaling cascades underlying long term potentiation (LTP). This open channel can then be blocked by uncompetitive antagonists such as ketamine, which enter the pore and inhibit ion flow (32). The impact of ketamine administration on memory is discussed in the clinical and ethical considerations section.

The Disinhibition Hypothesis (33) suggests that ketamine preferentially blocks NMDARs on GABAergic interneurons in the medial prefrontal cortex, reducing inhibitory control over pyramidal neurons (32). This disinhibition leads to increased glutamate release from pyramidal neurons, which in turn stimulates downstream neurons, enhancing synaptic plasticity and producing rapid antidepressant effects (30, 34).

#### 5.1.2 Glutamatergic AMPA receptors

Another key downstream effect of this heightened glutamate transmission is the acute activation of α-amino-3-hydroxy-5-methyl-4-isoxazole propionic acid receptors (AMPARs)—ionotropic glutamatergic receptors responsible for fast excitatory synaptic transmission and synaptic plasticity (30, 34, 35). Prior studies have demonstrated that co-administering AMPAR agonists with ketamine enhances its antidepressant effects, while blocking AMPARs with the antagonist 2,3-dioxo-6-nitro-7-sulfamoyl-benzo[f]quinoxaline (NBQX) prevents ketamine’s ability to alleviate depressive symptoms in mouse models, as observed in multiple behavioral assays (32). Notably, within three hours of ketamine administration via injection, the GluA1 and GluA2 AMPAR subunits are upregulated in the hippocampus and medial prefrontal cortex, suggesting a mechanism of enhanced synaptic strengthening (36). Consistent with this, *in vivo* electrophysiological studies in mice have shown that ketamine increases AMPAR-mediated synaptic transmission in the medial prefrontal cortex and in the CA1 and CA3 subregions of the hippocampus (32). Collectively, these findings highlight AMPAR activation as a critical component of ketamine’s rapid and sustained antidepressant effects.

Brain-derived neurotrophic factor (BDNF) is a growth factor synthesized in the brain that plays an important role in neurogenesis, neuroprotection, brain development, and synaptic plasticity (30). Studies in mice (37) have shown that ketamine administration significantly increases BDNF protein levels within 30 minutes. This occurs when the glutamate binds to and activates AMPARs, causing postsynaptic depolarization and calcium influx, which triggers fusion of BDNF-containing vesicles and rapid activity-dependent BDNF release into the synaptic cleft. Additionally, by blocking spontaneous NMDAR activity on postsynaptic pyramidal neurons, ketamine inhibits eukaryotic elongation factor 2 kinase (eEF2K), leading to dephosphorylation of eukaryotic elongation factor 2 (eEF2), lifting translational suppression and enabling rapid new BDNF synthesis. BDNF is essential for ketamine’s antidepressant effects, as ketamine fails to produce these effects in mice with a *Bdnf* gene knockout. Similarly, human patients carrying a Val66Met single-nucleotide polymorphism (SNP) in the BDNF gene, which impairs BDNF processing and secretion, do not respond to ketamine’s antidepressant actions (32, 38). These results indicate that an increase in BDNF synthesis is required for the antidepressant effects of ketamine.

Increased BDNF synthesis stimulates both the phosphatidylinositol 3-kinase (PI3K) and mitogen-activated protein kinase (MEK-MAPK) pathways, both of which promote activation of mechanistic target of rapamycin complex 1 (mTORC1) (32, 38). In the PI3K pathway, activation of PI3K leads to the phosphorylation and activation of protein kinase B (PKB/Akt). Activated PKB then inhibits tuberous sclerosis complex 2 (TSC2), which is a negative regulator of mTORC1. This inhibition of TSC2 allows mTORC1 to be activated (30). In the MEK-MAPK pathway, activation of MEK results in the activation of MAPK/ERK (extracellular signal-regulated kinase). ERK can then phosphorylate the Raptor (regulatory-associated protein of mTOR) scaffold protein in the mTORC1 complex, leading to mTORC1 activation. mTOR ultimately controls neurogenesis, protein synthesis, and dendritic spine growth by phosphorating p70S6 kinase and repressing 4E binding proteins (4EBPs). Excessive or uncontrolled neurogenesis can lead to tumor formation, and the long-term effects of repeated ketamine use on neural proliferation and cancer risk remain uncertain. Multiple studies have demonstrated that ketamine rapidly activates the vital mTOR pathway in the prefrontal cortex and hippocampus of rodents within 30 minutes (32, 34). This mechanism is thought to induce long-lasting changes in synaptic plasticity and contribute to ketamine’s overall prolonged antidepressant effects.

#### 5.1.3 Direct inhibition hypothesis

An alternative proposed mechanism for ketamine’s antidepressant effects is the direct inhibition hypothesis (34, 39). This hypothesis suggests that ketamine directly inhibits extra-synaptic GluN2B-containing NMDARs, which are located on dendrites adjacent to glial cells (34). These receptors are tonically activated by low levels of extracellular glutamate. Under normal conditions, this constant activation suppresses protein synthesis via the mTOR pathway to maintain synaptic homeostasis. By blocking extra-synaptic GluN2B-NMDARs, ketamine relieves this suppression, allowing mTOR-dependent protein synthesis and synaptic plasticity to increase, which contributes to its rapid antidepressant effects (32, 39). Support for this hypothesis comes from studies showing that ketamine fails to reduce depressive behaviors in mice lacking GluN2B-containing NMDARs. Additionally, selective GluN2B antagonists, such as traxoprodil, exhibit antidepressant-like effects similar to ketamine in mouse models, although with a slower onset (32, 34, 39).

Ketamine also produces changes in dopaminergic signaling within mesolimbic pathways (40). However, the role of these effects in ketamine’s reinforcing and addictive potential is not fully understood. Ketamine dependence appears to be driven largely by psychological factors, with its dissociative and psychotomimetic effects contributing to its potential for misuse (41).

### 5.2 Neurocircuitry and default mode network effects

Numerous ketamine studies have demonstrated that the effects of this drug are not solely at the receptor-level, but also at the large-scale brain network level, particularly within the Default Mode Network (DMN) (42–44). The DMN is mainly comprised of the medial prefrontal cortex, precuneus, anterior cingulate cortex, posterior cingulate cortex, and angular gyrus, and is known to be hyperconnected in major depressive disorder (MDD). This hyperconnectivity in the DMN is instrumental in explaining the negative self-focus and rumination found in MDD patients (45, 46). Thus, ketamine’s impact on DMN connectivity helps explain how it produces antidepressant effects.

Resting-state functional magnetic resonance imaging (rs-fMRI) research suggests that the impact of ketamine on the DMN is temporal and occurs over multiple phases. In healthy human subjects receiving S-ketamine, the pregenual anterior cingulate cortex (pgACC), an important node for emotional regulation in the DMN, displayed immediate increases in connectivity with the medial and dorsomedial prefrontal cortex, even while local glutamate levels remained low. Connectivity was subsequently measured twenty-four hours later and showed a very different pattern. Specifically, the connectivity decreased between the pgACC and both the dorsolateral prefrontal cortex and inferior parietal lobe, while glutamate levels rose, reflecting homeostatic network rebalancing (45). In short, then, the data indicated that while early increases in pgACC connectivity occurred even when glutamate levels were initially low, over time, glutamate levels increased, suggesting that these early network changes may drive subsequent neurochemical adaptations (45).

In patients with TRD, ketamine’s effects on DMN connectivity appear to significantly alleviate symptoms of depression, and the strength of DMN connectivity may help predict which patients are most likely to respond. Utilizing fMRI, Wade et al. (46) demonstrated that patients with stronger connectivity at baseline between the anterior DMN and the posterior insula predicted greater symptom improvement following a series of four ketamine infusions. Additionally, Zhang et al. (47) reported that patients with stronger baseline connectivity between the DMN and the hippocampus were more likely to achieve remission of depression symptoms. This study further reported that lower DMN-hippocampus connectivity after ketamine treatment corresponded with reduced depressive symptoms, indicating that the treatment promoted more flexible, adaptive network patterns. Both these studies support the idea that stronger or more rigid baseline network patterns allow for greater therapeutic reorganization.

Beyond simply changing the average strength of connections, the research shows that ketamine also alters how brain networks can become more flexible, allowing them to shift and interact more dynamically over time. After administering ketamine to patients, co-activation analyses show that these patients spend less time in the visual network states and more time in the central executive network states. These patients also demonstrated a greater number of transitions from the salience network to the central executive network (48). Fundamentally, the findings suggest that ketamine helps patients’ brains move from rigid, inward-focused networks toward more flexible and goal-oriented states.

Overall, these studies suggest that the DMN may serve not only as a key target of ketamine but also as a potential biomarker that could help predict which patients are most likely to benefit from this treatment, while also promoting brain networks that are flexible and goal directed. These findings support a coordinated cascade model in which NMDA receptor blockade produces an early glutamate surge, and AMPA receptor activation triggers BDNF and mTOR-dependent synaptogenesis. These cellular effects markedly reconfigure the DMN and related networks. Network stabilization allows for more adaptive and flexible connections that correlate with clinical improvement. Overall, ketamine reshapes and dynamically restores flexibility to pathological network patterns, providing a mechanistic basis for its rapid and powerful antidepressant effects.

## 6 Clinical uses of ketamine

### 6.1 On-label or prescription uses of ketamine—anesthesia, acute pain management, treatment-resistant depression

As discussed above, racemic ketamine (Ketalar), approved by the FDA in 1970, was originally developed for use as either the sole anesthetic agent for diagnostic and surgical procedures, or utilized in conjunction with other by anesthetics, such as nitrous oxide. Beginning in the early 1970s, ketamine was used by both veterinarians and anesthesiologists in the operating room to sedate patients. In contrast to other types of anesthetics, ketamine had “dissociative properties,” which is why it is sometimes referred to as “*dissociative anesthesia”* (7) that placed patients into a trance-like state, separating them from their reality while blocking their pain. Specifically, the anesthetic properties of ketamine function by blocking N-methyl-D-aspartate (NMDA) receptors in the brain, preventing the patient from perceiving pain, and inducing a dissociative state. At the same time, ketamine provided sedation and relief from pain but did *not* produce substantial respiratory depression as did some other anesthetics (7, 9). Ketamine was characterized as a “*unique drug*” in that it produced three effects simultaneously: its analgesic or pain-relieving effects, sedative effects, and its amnesic effects or short-term memory loss (49). In short, given these factors, ketamine proved to be particularly useful in emergency room settings as well as in the field (on battlefields, during times of war, i.e., Vietnam) (7, 50).

In addition to its anesthetic applications, the FDA also approved ketamine for acute pain management. This drug is particularly useful in emergency rooms, in operating rooms and in post-op where ketamine is given to the patient in lower doses in an effort to diminish the pain without completely sedating the patient (6, 7, 49, 51–53).

Moreover, in 2019, the FDA approved the drug, *esketamine* (a more potent version of ketamine) for use with treatment-resistant depression or TRD. Although the drug had been used for several years off-label to treat depression, it did not earn approval from the FDA until a few years ago. Groundbreaking research studies on esketamine (29, 54–56) demonstrated that while patients taking traditional anti-depressants did not see results for weeks to several months, their studies on esketamine demonstrated fast-acting results, sometimes within a few hours (14, 15, 17, 57–60). In contrast to conventional antidepressants that target dopamine, norepinephrine, or serotonin systems, esketamine antagonizes the NMDA receptors, increasing synaptic plasticity, thereby culminating in rapidly improved mood levels and increased cognitive abilities (19, 60). Additionally, it has been demonstrated that ketamine in conjunction with intensive psychotherapy is yielding promising results for the treatment of TRD (61).

Esketamine, marketed as “Spravato,” is a nasal spray used combined with oral antidepressants to treat patients who have not benefited from traditional antidepressants alone. Due to the risks associated with dissociation, sedation, and potential misuse/abuse, Spravoto is administered under strict clinical conditions. Patients administer the nasal spray to themselves but do so under supervision from medical personnel in a clinical setting. Patients are monitored throughout, and clinicians decide when it is safe to leave the clinic/office (16–19, 59).

In 2020, the FDA approved esketamine, co-administered with an oral antidepressant, for the treatment of depressive symptoms in adults with major depressive disorder (MDD) presenting with acute suicidal ideation or behavior. Studies (29, 55, 62) demonstrated that esketamine, given in context within the context of comprehensive care, produced a rapid reduction in overall depressive symptom severity with 24 hours in high-risk patients, especially in those patients who are high-risk and had a history of suicide attempt or forms of self-harm (55). However, esketamine has not been shown to independently prevent suicide or directly reduce suicidal ideation, and its use does not preclude the need for clinical hospitalization if indicated. Further investigation in larger, longitudinal cohorts is required to confirm these preliminary outcomes and establish its long-term clinical role. Mechanistically, esketamine functions to promote neural connectivity, synaptic repair, and growth and helps to restore brain functioning and mood stabilization, previously crippled by serious depression (62).

### 6.2 Off-label psychiatric uses of ketamine—PTSD, anxiety disorder and bipolar disorder

In addition to the FDA approved uses of ketamine and its derivatives, in recent years, researchers have found that this drug is useful in treating various mental health conditions.

One such off-label psychiatric use of ketamine has been for the treatment of post-traumatic stress disorder (PTSD). Specifically, research studies (13, 23, 24, 63–69) demonstrate that intravenous (IV) racemic ketamine significantly reduces PTSD symptoms by blocking pain receptors in the brain and calming the HPA axis. In addition, this drug also functions to create healthy new synapses in the brain. Other studies (26, 68) have demonstrated that IV racemic ketamine enhances neuroplasticity of the brain, allowing for individuals to process their post-traumatic situations in a more constructive manner. These studies also show that ketamine is a form of preventative treatment for PTSD, as it interferes with fear memory formation. For example, a recent research study in mice (70) showed that locally infused ketamine into the nucleus accumbens immediately after a traumatic event impaired the storage of fear memories. This effect is linked to ketamine’s action on NMDA receptors, which play a key role in strengthening synapses between glutamatergic neurons—an essential process in memory storage (71). By enhancing neural plasticity, ketamine could help individuals unlearn fear-based memories and replace them with safer associations. Studies have demonstrated that early intervention with racemic ketamine may also prevent traumatic memories from becoming long-term (24, 72).

A second off-label psychiatric use of ketamine has been to treat social anxiety disorder (SAD) and generalized anxiety disorder (GAD). One of the major problems associated with conventional anxiety medications is that they often lead to addiction/dependence or sedate patients, making it difficult for them to carry out daily activities. However, with respect to ketamine, recent studies using different dosing regimens and routes of administration, have shown that it reduced anxiety symptoms very quickly without causing addiction/dependence upon the medication (20–22, 73, 74). According to these studies (74, 75), as little as one intravenous dose of ketamine can fully alleviate symptoms of anxiety for several days, up to several weeks. Although the data yielded from these early studies seems promising in providing relief from social and general anxiety disorder, future research is warranted with larger samples over an extended time period.

A third off-label psychiatric use of ketamine has been to treat bipolar disorder (28, 76–78). Whereas much research has been conducted on major depressive disorder yielding promising results, relatively little research has been conducted on ketamine and bipolar disorder. Given that bipolar disorder is a complex psychiatric condition, researchers have found that there is no “easy fix” (27, 79, 80). Researchers in their studies (25, 26, 28) have found that there is *weak* to *moderate* evidence of esketamine being a promising treatment for bipolar disorder (26). Other studies (28, 81, 82) have demonstrated that intravenous ketamine leads to decreases in suicidal tendencies, anhedonia (lack of joy/emotional flatness), and functions to alleviate anxiety. Moreover, its mood-stabilizing properties are also hypothesized in these studies. Preliminary research findings (28, 83) indicate that ketamine, used in conjunction with other conventional oral bipolar medications, along with psychotherapy reduced symptoms of bipolar disorder. However, there is a need for additional research studies that focus on longer-term outcomes, repeated acute and maintenance infusions using randomized and controlled study designs, and studies using alternative formulations of esketamine, arketamine (sublingual, nasal spray, IV, subcutaneous) (79, 84). The current psychiatric applications of ketamine are summarized in **Table 1**. Additionally, FDA-approved and off-label psychiatric uses of ketamine are summarized in **Table 2**.

**Table 1:** Summary of current psychiatric applications of ketamine.

Psychiatric indication	Evidence level	Ketamine formulation /route	Regulatory status	Key clinical findings	Major limitations	Guideline/clinical position
Treatment-resistant depression (TRD)	High	IV racemic ketamine; intranasal esketamine	Esketamine FDA-approved; IV off-label	Rapid antidepressant effects within hours to days; maintenance therapy may prolong benefit	Need for repeated dosing; long-term safety remains under study	Recommended for appropriate patients with monitoring
Major depressive disorder with acute suicidal ideation	High	Intranasal esketamine; IV ketamine	Esketamine FDA-approved for specific indication; IV off-label	Rapid reduction in suicidal ideation when used with standard care	Benefit may diminish over time; ongoing treatment often required	Use in conjunction with comprehensive psychiatric management
Bipolar depression	Moderate	IV ketamine	Off-label	Rapid antidepressant effects reported in selected studies	Limited long-term efficacy and safety data	Consider in carefully selected patients
Post-traumatic stress disorder (PTSD)	Moderate	IV ketamine	Off-label	Improvement in PTSD symptoms demonstrated in early trials	Optimal dosing strategy remains uncertain	Promising but investigational
Anxiety disorders	Low–Moderate	IV ketamine	Off-label	Short-term reduction in anxiety symptoms	Limited high-quality randomized trials	Not routine clinical practice
Other psychiatric disorders	Emerging	Various	Off-label	Early reports suggest possible benefit in selected neuropsychiatric conditions	Insufficient evidence for routine use	Further research required

**Table 2:** FDA-approved and off-label psychiatric uses of ketamine.

Indication	Formulation	Regulatory status	Evidence	Clinical considerations
TRD	Intranasal esketamine	FDA-approved	Strong	REMS required
TRD	IV racemic ketamine	Off-label	Strong	Specialty clinics
Acute suicidal ideation	Intranasal esketamine	FDA-approved (specific indication)	Strong	Adjunct to standard care
PTSD	IV ketamine	Off-label	Moderate	Emerging evidence
Bipolar depression	IV ketamine	Off-label	Moderate	Limited long-term data
Anxiety disorders	IV ketamine	Off-label	Limited–Moderate	Further trials needed

## 7 Recreational uses and misuses of ketamine

Ketamine, or Special K, Ket, Purple, Green K, Cat killer, Kit Kat, Super C, Jet or Super Acid as it is variously referred to on the streets, has, and is, being used as a party drug among young people. It is often used by individuals at nightclubs, bars, concerts, raves, or other events. As Halkitis et al. (85), note, ketamine is one of the prevalent drugs used in clubs (in addition to (drugs such as ecstasy, powdered cocaine, GHB, and methamphetamines) as part of their *“polydrug experience.”* In addition, research shows that it is also a popular drug among high school and college students (86, 87). Party drugs like ketamine often contain psychoactive substances that distort the person’s perception of reality. Recreational users of ketamine are attracted to this drug because it gives them a sensation of “floating” or detachment (88, 89). Many users talk about being in a “*dreamlike state*” when under the influence of ketamine. Given that Ketamine possesses the ability to sedate, cause short-term memory loss, and immobilize individuals, sadly, it has been used in countless non-consensual sexual encounters—which is why it has been referred to as the “*date rape drug*” (90).

Ketamine can be administered in the following ways: in powder form, it can be snorted; it can be injected; it can be smoked or even mixed into beverages (52). Once in the body, ketamine works very quickly to cause sleep, inhibit memory, and gives a floating sensation or high. If the drug is snorted or injected, users begin to feel the effects within minutes (91).

In terms of usage, according to research (86, 89, 92, 93), ketamine use is increasing among the younger population. As Palamar et al. (92, 93) document, between 2017 and 2022, ketamine use among this segment of the population increased by over 350%. From 2021-2022, recreational use of ketamine increased by 40% from the previous year (92). Similarly, during this same time period, seizures of this drug within the United States also increased from 57.8 kg to 703.3 kg (93). Statistics demonstrate that global seizures of ketamine peaked in 2022 at 33.9 tons, particularly in East and Southeast Asia (At this time, however, there were also large seizures in the Middle East, North America, Western and Central Europe, as well as South Africa and the Caribbean (94).

Analysis of the Global Drug Survey 2018 found that 5.93% of respondents reported having used ketamine at least once in their lifetime (95). While its use and abuse are more widespread throughout East and Southeast Asia (8.2% of the population) (88, 94, 96, 97), in the United States, statistics show that over 3 million or approximately 1% of Americans reported having used ketamine at least once, recreationally (98). The majority of users were between 12 and 25 years of age, with the typical user being a male around 25 years of age (99).

According to the Monitoring the Future study conducted at the University of Michigan, approximately 1.1% of high school seniors in the United States reported using ketamine in the past year (87). Some of these students reported trying this drug for “fun” but others reported using it to manage depression (86, 92).

## 8 Clinical considerations, and guidelines

### 8.1 Clinical and ethical considerations

Although ketamine has significant *positive effects* on human physiology, most notably, its rapidly acting antidepressant abilities, its acute and chronic use can also cause some serious adverse effects in patients.

Turning firstly to the effects of acute administration of this drug, at the cardiovascular level, ketamine can lead to tachycardia and hypertension which can harm patients with weakened cardiovascular systems, in particular, those with heart failure (100–102). Ketamine administration is therefore, contraindicated in patients with uncontrolled hypertension and other uncontrolled cardiovascular conditions, who are pregnant or breastfeeding, have unmanaged schizophrenia or psychosis, or have active substance abuse (103). Another side effect of acute administration of ketamine, at the psychological and perceptual levels, is the feeling of dissociation or detachment from the body, vivid dreams, hallucinations, confusion, anxiety and agitation—conditions which are perceived by some patients to be disturbing (104, 105).

A third negative effect of acute ketamine administration centers on various neurological and physical issues. Administration of this drug can cause such conditions as blurred vision, loss of motor coordination or ataxia, rapid eye movement and dizziness (106, 107).

Turning to chronic adverse effects of ketamine administration, one such effect is neurological and cognitive impairment. Specifically, long-term use has been linked to deficits in memory, attention, and executive functioning in conjunction with structural changes in the brain. Specifically, research has shown that long-term use has led to reductions in gray matter volume across the frontal, parietal and occipital lobes. In addition, its usage has been associated with reduced white matter integrity, which may disrupt connectivity between frontal and temporoparietal brain regions (108–110).

Chronic ketamine administration has also been shown to induce psychotic-like symptoms, persistent paranoia, mood instability, long-term dissociative states and delusional thoughts (108, 111).

Systemically, chronic use of ketamine has also been associated with medical issues such as shrinking bladder capacity, blood in the urine, and severe pelvic pain. Its use can also cause severe inflammation and ulceration of the bladder (ketamine-induced cystitis) which has irreversible urinary tract damage, pain and incontinence, possibly leading to kidney failure (112). Additionally, chronic ketamine use can cause gastrointestinal issues such as nausea, vomiting, and severe, recurring abdominal pain or K-cramps as well as abnormal liver and gallbladder function (112, 113).

Further, long-term usage can lead to tolerance and psychological addiction, strong drug cravings, depression, and anxiety during periods of withdrawal or non-use. Specifically, research has shown that chronic administration of ketamine causes dependence and addiction through the disruption of glutamatergic and dopaminergic neurotransmission in subcortical reward regions, such as in the nucleus accumbens (108, 114–116).

### 8.2 Guidelines

Given the potential for ketamine to cause serious adverse effects on patient health, clinical and ethical guidelines have been established to regulate its administration. The FDA Risk Evaluation and Mitigation Strategy (REMS) for intranasal esketamine (Spravato) was implemented due to the serious concerns of dissociation, cardiovascular effects, sedation, and potential for abuse. The REMS program calls for esketamine to be administered in special certified healthcare clinics, under the direct supervision of a healthcare provider, and to be monitored by a healthcare provider for two hours post-administration. Esketamine is strictly prohibited from being administered at home under the REMS. The starting dose of esketamine is either 56 mg or 84 mg intranasally. The patient is monitored for changes in blood pressure and respiration, as well as any differences in neuropsychiatric function. Providers are additionally required to report adverse events, which supports ongoing safety monitoring under the REMS program (117). While the REMS program benefits the safety of patients, it also produces some ethical and clinical challenges. One such challenge that the program presents is the increased workload of the healthcare team due to the long administration and monitoring timeframe. Additionally, patients from rural and medically underserved areas face restricted access to this treatment. The restrictions of the REMS program worsen the mental healthcare disparities in medically underserved areas for people who need this treatment and may have to find other treatment that may be less effective. Lastly, the REMS program only applies to esketamine and not to racemic ketamine or arketamine. This creates challenges for both clinicians and patients by introducing inconsistencies in regulatory oversight, and monitoring guidelines. Safety considerations for ketamine administration in psychiatry are summarized in **Table 3**.

**Table 3:** Safety considerations for ketamine administration in psychiatry.

Safety domain	Clinical considerations	Risk mitigation
Acute adverse effects	Dissociation, sedation, nausea, transient BP/HR increases	Monitor during administration
Psychiatric adverse effects	Psychotomimetic symptoms, anxiety	Careful patient selection
Repeated/long-term treatment	Limited long-term safety; cognitive concerns	Periodic reassessment
Misuse/dependence	Tolerance, diversion, ketamine use disorder	Screening and controlled dispensing
Urinary/systemic toxicity	Chronic misuse associated with cystitis	Avoid prolonged misuse; educate patients
Telehealth	Reduced supervision, diversion risk	Standardized protocols and oversight
Clinical implementation	Patient selection, monitoring, informed consent	Follow evidence-based guidelines
Ethical/regulatory	Off-label use, commercialization, REMS	Adhere to regulations and professional guidance

## 9 Telehealth ketamine boom with oral/sublingual prescribing

During the COVID-19 pandemic regulations were relaxed that temporarily allowed for the prescribing of controlled substances by healthcare professionals without an in-person examination. The rapid expansion of telehealth clinics this period led to a dramatic rise in clinicians prescribing oral or sublingual racemic ketamine off-label for home administration, commonly in the form of lozenges (118). While this situation created greater access to ketamine treatment, especially for patients in medically underserved areas, it has also raised concerns centering around safety and monitoring, as these at-home administrations occur outside the REMS framework. Currently, standardized protocols for oral ketamine administration do *not* exist, which has led to variability in dosing, frequency, and monitoring among clinicians. Importantly, there is still limited evidence that oral ketamine is effective for treating depression and there has been only a paucity of high-quality clinical trials investigating oral ketamine. Pacilio et al. (119), in a nationwide survey conducted in the United States, reported that 43.5% of outpatient ketamine clinics provided at-home oral/sublingual ketamine. This study demonstrates the urgent need for standardized dosing, careful monitoring, and clear regulatory oversight to ensure patient safety for all forms of ketamine, not just esketamine.

## 10 Discussion

### 10.1 Key clinical challenges

Even though this review highlights the benefits of ketamine usage, nevertheless, there are still many challenges facing the landscape of ketamine administration, research, patient outcomes, and safety.

Given that ketamine administration is associated with distinct acute and chronic effects, this drug warrants careful clinical assessment and monitoring on the part of the treating clinician. As was discussed earlier in this paper, acute administration may cause symptoms ranging from temporary increases in heart rate and blood pressure, vomiting and nausea, blurred vision, lack of coordination, dissociation, hallucinations, to disturbances in perception. Moreover, with prolonged exposure to ketamine, physicians also need to be cognizant of the potential for tolerance, psychological addiction, the development of urinary tract issues (such as cystitis, K-cramps, blood in the urine, abnormal gallbladder and liver function), along with neurological, cognitive impairment. Therefore, we would argue that, prior to administering this drug, clinicians should assess the patient’s baseline liver and kidney functions, take thorough cardiovascular and psychiatric histories, perform appropriate diagnostic testing, as well as reviewing concomitant medications and substance use. Further, careful consideration should be given to dosage and route selection and ongoing monitoring of the patient during administration of ketamine in an effort to detect any adverse effects or possible toxicity of the drug. We argue that precautions are especially important where ketamine is administered over the long term or outside of the clinical setting where complications may otherwise go undetected and untreated.

### 10.2 Limitations of current research

While the numerous ketamine clinical studies addressed in this paper have advanced our understanding of its antidepressant mechanisms, therapeutic potential, and the challenges associated with its clinical use, there are still significant methodological limitations that researchers face when conducting these trials (120). Given that ketamine has prominent dissociative and psychoactive effects, participants and researchers can often infer which treatment group a participant has been assigned to based on how they respond. This clearly introduces *expectancy bias* into the studies, making it more difficult to validly assess ketamine’s efficacy. Some studies have utilized an *active placebo*, such as midazolam, to minimize expectancy bias by more closely mimicking ketamine’s acute sedative and perceptual effects (121). A recent rigorously conducted 2025 KARMA-Dep 2 study reported no significant differences in outcomes between repeated ketamine infusions and midazolam (122). Therefore, while active placebos improve blinding and reduce expectancy effects, they also make it harder to detect clear differences between ketamine and the control treatment. Distinguishing ketamine’s true therapeutic effects from placebo responses is a significant challenge that must be resolved to advance research and clarify its efficacy. Lastly, there is still significant variability (no standardization) in the design of ketamine clinical trials, the various safety protocols employed, and their methodological rigor, which limited comparisons and conclusions that can be drawn across studies.

While most ketamine clinical trials focus on short-term effects of administration, much remains unknown on the long-term efficacy and safety. Long-term safety and efficacy data are limited for adolescents and older adults, which provides clinicians with little guidance on appropriate dosing and monitoring for these age populations. Additionally, there is limited research on oral racemic ketamine, and no standardized guidelines exist for its dosing, frequency, or safety. Although esketamine has comparatively more long-term safety and efficacy data due to its regulatory approval, long-term safety data for racemic ketamine remain scant. This raises concerns about the potential long-term cumulative effects of repeated racemic ketamine, such as on cognition, cardiovascular health, and urinary function. More clinical research is warranted to support the growing use of oral racemic ketamine for depression to ensure long-term patient safety.

The rapid expansion of telehealth ketamine clinics has allowed for greater access to at-home oral ketamine, particularly for patients in rural or underserved areas. However, much of this prescribing occurs with minimal regulatory oversight. Patients in rural or medically underserved often lack access to supervised ketamine administration, which in turn disproportionately exposes them to the less regulated practice at-home oral ketamine administration. This obviously raises concerns about patient safety, consistent monitoring, and timely reporting of adverse events. The lack of standardized dosing protocols for oral racemic ketamine and protocols for follow ups following adverse events worsen the risks for patients. There is a clear urgent need for the creation of clear clinical guidelines and regulations for the administration of oral racemic ketamine. Ideally, a REMS protocol would be created by the FDA for oral racemic ketamine.

## 11 Conclusions and future directions

Overall, this synthesis highlights the expanding clinical role of ketamine, while also bringing into focus several ongoing operational and clinical challenges. This review followed a structured search strategy adhering to PRISMA 2020 guidelines to ensure a rigorous and transparent methodology. Interpreting these collective findings, however, requires considering the broad structural diversity of the current literature, where variations in dosing, endpoints, and study designs made a narrative synthesis the most appropriate approach to capture the field. While pairing clinical trials with observational and mechanistic literature offered a thorough perspective, it inevitably paired controlled efficacy data with more tentative, real-world findings and familiar issues like unblinding. Recognizing these varying levels of evidence helps delineate clear priorities for future investigation.

Future research on ketamine for psychiatric disorders should prioritize strengthening the evidence base and refining its clinical implementation. A central objective needs to focus on conducting large-scale, randomized, controlled trials evaluating the long-term safety and efficacy of repeated ketamine administration for major depressive disorder (MDD). Particular attention must be paid to the durability of antidepressant response, potential cognitive sequelae, and the risk of misuse, tolerance, or dependence associated with ongoing treatment. Many existing studies have been limited by small sample sizes, short follow-up durations, and restricted population diversity, thereby constraining generalizability. Accordingly, future investigations should employ adequately powered, methodologically rigorous designs with extended follow-up periods and demographically representative samples.

Standardization of treatment protocols is also essential. Substantial heterogeneity persists across studies with respect to dosing strategies, frequency and duration of administration, and routes of delivery. Comparative effectiveness trials are needed to determine optimal dosing regimens and to evaluate differences among intravenous (IV), intranasal, sublingual, intramuscular, and oral formulations. Furthermore, systematic examination of ketamine administered in conjunction with structured psychotherapeutic interventions may clarify whether combined approaches enhance and sustain therapeutic benefit. Establishing standardized clinical guidelines will require harmonization of these variables across future trials.

Given the increasing availability of at-home oral racemic ketamine, rigorous evaluation of its safety profile, efficacy, and potential for diversion or misuse is warranted. Prospective studies and post-marketing surveillance efforts should assess adherence, monitoring frameworks, and risk mitigation strategies in real-world settings.

Biomarker development represents another critical area for advancement. Identification and validation of predictive biomarkers—including neuroimaging measures such as functional connectivity on functional magnetic resonance imaging (fMRI), as well as molecular, genetic, and inflammatory markers—may facilitate patient stratification and personalized treatment approaches. Parallel mechanistic research should further elucidate ketamine’s modulation of glutamatergic neurotransmission, particularly its effects on N-methyl-D-aspartate (NMDA) and α-amino-3-hydroxy-5-methyl-4-isoxazolepropionic acid (AMPA) receptor signaling, synaptic plasticity, and neurotrophic pathways. Such insights may inform the development of novel rapid-acting antidepressants with improved safety and tolerability profiles.

Beyond MDD, future studies should systematically evaluate ketamine’s therapeutic potential across additional psychiatric conditions, including anxiety disorders, PTSD, and substance use disorders. Investigations of combination strategies with these other disorders—such as ketamine administered alongside cognitive behavioral therapy, transcranial magnetic stimulation, or electroconvulsive therapy—may further clarify its role within multimodal treatment frameworks.

Finally, the establishment of national and international patient registries is recommended to systematically collect longitudinal real-world data on treatment outcomes, adverse effects, and patterns of use across diverse clinical settings. Such infrastructure would support pharmacovigilance efforts, inform evidence-based guidelines, and promote the responsible and equitable integration of ketamine into psychiatric practice.

## Data Availability

The original contributions presented in the study are included in the article/supplementary material. Further inquiries can be directed to the corresponding author.
